# Sigma1 Receptor Modulates Plasma Membrane and Mitochondrial Peroxiporins

**DOI:** 10.3390/cells14141082

**Published:** 2025-07-15

**Authors:** Giorgia Pellavio, Giorgia Senise, Chiara Pia Vicenzo, Umberto Laforenza

**Affiliations:** 1 Department of Molecular Medicine, Human Physiology Unit, University of Pavia, 27100 Pavia, Italy; giorgia.pellavio@unipv.it (G.P.); giorgia.senise01@universitadipavia.it (G.S.); vicenzochiarapia@gmail.com (C.P.V.); 2 Centre for Health Technologies (CHT), University of Pavia, 27100 Pavia, Italy

**Keywords:** aquaporins, mitochondrial permeability, HyPer7 biosensor, oxidative stress, hydrogen peroxide, HeLa

## Abstract

Sigma1 receptor (S1R) and some aquaporins (AQPs) are involved in controlling oxidative stress, but only recently has their possible interaction emerged. S1R acts by interacting with proteins in the plasma membrane and organelles and AQPs by favoring the hydrogen peroxide (H_2_O_2_) cell removal. To date, the possible regulation of peroxiporins by S1R has not been explored. Using H_2_O_2_ HyPer7 biosensors and knockdown techniques, we investigated (1) the AQPs and S1R functional involvement in H_2_O_2_ diffusion through the plasma membrane and in the outer and inner mitochondrial membranes, and (2) the possible interaction between S1R and AQPs. Our data showed the functional involvement of different AQPs in the diffusion of H_2_O_2_: AQP3, AQP6, and AQP8 in the plasma membrane; AQP6 in the outer mitochondrial membrane; and AQP6 and AQP8 in the inner mitochondrial membrane. The knockdown of S1R demonstrated its involvement in the overall diffusion of H_2_O_2_ across the three compartments. The double knockdown of S1R and a single AQP indicated that AQP8 and AQP6 could be regulated by S1R. These findings demonstrate the coordinated role of AQPs in the mitochondria and the plasma membranes and that S1R modulates the AQP-facilitated H_2_O_2_ cell removal, thus controlling the oxidative status and, most likely, the oxidative stress.

## 1. Introduction

The sigma1 (S1R) and sigma2 receptors are enigmatic proteins. When discovered, they had been framed as opioid receptors, but later on, they were shown to have pharmacological differences. Unlike canonical opioid receptors, the sigma receptors have a divergent ligand binding profile: they have no affinity for naloxone, naltrexone, and (−) benzomorphans, while they bind with high affinity to (+)-enantiomers [1,2].

S1R functions mainly as a ligand-operated chaperone that modulates signaling pathways inside the cell. Agonists and antagonists were synthesized for this receptor, but to date, no endogenous ligand has been identified with certainty. In 1996, S1R was cloned, and curiously, its amino acid sequence was found to be different from known mammalian proteins [3]. The closest homolog of the S1R is a sterol isomerase of yeast; however, the S1R has no sterol isomerase activity [3].

In the resting state, it is localized intracellularly in the mitochondrion-associated endoplasmic reticulum membrane (MAM). Upon activation, S1R translocates to or interacts with more than 49 different proteins, particularly G protein-coupled receptors, and ion channels, modulating their function (for a complete review, see [4]). The interaction/translocation can occur with proteins localized in the endoplasmic reticulum but also with proteins localized at the mitochondrial, nuclear, and plasma membrane levels. Among the functions of S1R, we can include lipid transport and metabolism, modulation of ion channels, intracellular calcium homeostasis, and control of the cellular oxidative state [5]. Moreover, S1R is involved in many pathologic conditions characterized by high oxidative stress, such as neurodegenerative diseases (amyotrophic lateral sclerosis and Alzheimer’s disease) and different cancers [6,7]. For these reasons, SRs are considered promising therapeutic targets, and different ligands have been developed. In particular, S1R modulators are under investigation for treating several diseases involving oxidative stress [8].

Recently, we studied S1R agonists and antagonists as potential aquaporin (AQP) regulators [9,10]. AQPs are water channel proteins belonging to the major intrinsic proteins superfamily that favor the diffusion of water and small molecules through the cell membranes. Some of them show additional hydrogen peroxide (H_2_O_2_) permeability and represent the sub-family of the peroxiporins: AQP0, 1, 3, 5, 6, 8, 9, and AQP11 [11,12,13,14,15,16,17,18,19,20,21,22]. Peroxiporins play a key role in ensuring ROS scavenging, and they are considered an antioxidant system. Variations in the permeability of these AQPs reflect the cellular oxidative state. It has been observed that oxidative stress reduced the permeability of peroxiporins to water and H_2_O_2_ with a negative feedback mechanism, worsening the oxidative stress itself, impairing normal cell functions, and leading to cell death [13]. Conversely, oxidative stress increased AQP-mediated permeability to water and H_2_O_2_ of mesothelioma cells by positive feedback, making these cells resistant to apoptosis [22]. Therefore, the possibility of regulating the peroxiporin-mediated H_2_O_2_ permeability seems important to control cell signaling and survival during oxidative stress [13,22,23]. Small molecules, heavy metal ions, antibodies, and nanoparticles were shown to modulate the permeability of AQPs [24,25].

In this scenario, we studied the effect of different S1R ligands on the AQP-mediated water and H_2_O_2_ permeability [10]. The well-established S1R agonists PRE084 and RC33 and three S1R ligands developed by Collina’s Group [26] could protect HeLa cells from oxidative stress, whereas the S1R antagonist NE100 did not [10]. All the compounds, except for RC33, were able to counteract the oxidative stress of HeLa cells selectively knocked down for S1R, demonstrating that the antioxidant mechanism seems mediated by both S1R and AQP-mediated permeability. Moreover, double-label immunofluorescence experiments showed a partial colocalization and/or contiguity of S1R and some AQPs in HeLa [10]. As a whole, these results suggest, on the one hand, that S1R ligands can also modulate the gating of AQPs; on the other hand, S1R could interact and modulate the antioxidant function of AQPs.

Some intracellular AQPs are located in proximity to S1R. In particular, AQP8 and AQP11 are expressed in mitochondria [27,28] and endoplasmic reticulum (ER) [12], respectively, and S1R is mainly expressed in MAM [29]. Moreover, S1R can also promote the translocation of proteins to the plasma membrane [5]. Unfortunately, the existing literature lacks studies that unequivocally demonstrate a functional role for AQPs in the diffusion of H_2_O_2_ across the plasma membrane and both the outer and inner mitochondrial membranes. Furthermore, there is no direct experimental evidence of the existence of a modulation carried out by S1R on AQPs.

This study has a dual purpose: (1) to functionally demonstrate whether and which AQPs mediate H_2_O_2_ diffusion both at the plasma membrane and in the sequential diffusion of H_2_O_2_ across the two mitochondrial membranes and (2) to investigate the possible interaction between S1R and AQPs in eustress conditions.

We examined the role of all the peroxiporins previously identified in HeLa cells, AQP3, AQP6, AQP8, AQP11, and S1R, in facilitating H_2_O_2_ permeability across three different cellular compartments using selective gene knockdown and ultrasensitive hydrogen peroxide indicator HyPer7 targeted to the cytoplasm, to the mitochondrial intermembrane space, and to the mitochondrial matrix.

The results of the present study provide evidence that different AQPs are involved in the diffusion of H_2_O_2_ in the three compartments, and S1R modulates AQP8 both in the plasma membrane and in the inner mitochondrial membrane and AQP6 in the inner and in the outer mitochondrial membranes and probably also in the plasma membrane.

## 2. Materials and Methods

### 2.1. Cell Culture

HeLa cells (ATCC CCL-2™, Manassas, VA, USA) were cultured at 37 °C under 5% CO_2_ in Dulbecco’s modified minimal essential medium–high glucose, supplemented with 10% fetal bovine serum, 1% L-glutamine, 1% penicillin, and 100 µg/mL streptomycin.

### 2.2. Gene Knockdown

In this study, we investigated peroxiporins (AQP3, AQP6, AQP8, and AQP11), whose presence in HeLa cells has been confirmed both at the mRNA and protein levels through Western blotting and immunohistochemistry ([24] and present paper Figures S1, S3–S6).

HeLa cells with reduced S1R and AQP3, AQP6, AQP8, and AQP11 were obtained as previously described [10,24]. To knockdown AQP6, ON-TARGETplus Human AQP6 (363) siRNA-SMARTpool from Dharmacon™ was used (L-011579-00-0005, Dharmacon™, Horizon Discovery Group, Waterbeach, UK). HeLa cells with reduced AQP3, AQP8, and AQP11 were obtained using esiRNA from Merck (human AQP3, EHU071641; human AQP8, EHU034911; human AQP11, EHU037771; Merck, Milan, Italy). The S1R knockdown in HeLa cells was achieved using siRNA-SMARTpool from Dharmacon^TM^ (ON-TARGETplus SMARTpool Human SIGMAR1 siRNA; FE5LHUMANXX0005; Dharmacon™, Horizon Discovery Group, Waterbeach, UK). Negative controls were obtained by transfecting cells with EHUFLUC esiRNA that targets firefly luciferase or with ON-TARGETplus Non-targeting Pool. INTERFERin siRNA Transfection Reagent (#409-10, Polyplus transfection, Illkirch-Graffenstaden, France) was utilized to transfect HeLa cells with siRNA-SMARTpool or esiRNA. Following the manufacturer’s instructions, cells were transfected at the 50% confluency by adding a culture medium containing siRNAs and esiRNAs solution. Knockdown solutions were prepared by adding siRNA (20 nM final concentration) and esiRNA (25 nM final concentration) to 200 μL Opti-MEM™ Reduced Serum Medium (31985070; Thermofisher Scientific, Monza, Italy) and then mixed with 12 μL INTERFERin siRNA Transfection Reagent. Then, the knockdown solution was mixed with 2 mL of Opti-MEM™, added to HeLa cells, and incubated for 24 or 48 h at 37 °C. To demonstrate that the cells were successfully knocked down, qRT-PCR (for AQP1, AQP6, AQP8, and AQP11) and Western blot analysis (for S1R) were performed 48 h after transfection. Knockdown of AQP8 was tested after 24 h from transfection. The results are shown in Figures S2–S7.

A double knockdown of single AQP and S1R was performed by diluting the two siRNAs (40/45 nM in total) in Opti-MEM™ with INTERFERin siRNA Transfection Reagent to prepare the knockdown solutions. Successively, the knockdown solutions were mixed with the Opti-MEM™ and added to the cells as indicated above. Consecutive knockdown was performed in two days to respect the different incubation times for AQP8 and S1R knockdown.

### 2.3. RNA Isolation and Real Time RT-PCR

Extraction of total RNA from HeLa cells, cells mock-transfected, and AQP-knockdown was performed by using Qiazol (Qiagen, Milan, Italy). cDNA was obtained using MMLV Reverse Transcriptase M1701 (Promega, Milan, Italy) following the manufacturer’s instructions. qPCR was carried out with specific primers (Table S1) and QuantiFast SYBRGreen PCR Master Mix (Qiagen, Milan, Italy) by using StepOnePlus Real-Time PCR System (Life Technologies Italia, Monza, Italy). The amplification protocol was previously described [22]. In each qPCR reaction, the melting curve analysis was performed to determine the specificity of the amplicons. Measures were expressed as ΔCq, calculated by subtracting the Cq of the housekeeping gene from the Cq of the AQP gene, and it must be remembered that low ∆Cq values represent high transcript expression values. In experiments involving gene knockdown, the results were indicated as fold change:

Fold change: −2^(ΔCq, Control − ΔCq, siRNA)^

### 2.4. Western Blot Analysis

Knocked down and mock-transfected HeLa cells were homogenized with RIPA solution containing (final concentration) 150 mM NaCl, 0.5% sodium deoxycholate, 0.1% SDS, 0.1% Triton X-100, 50 mM Tris-HCl, pH 8, and the protease inhibitor cocktail cOmplete (cOmplete Tablets EASYpack, 04693116001; Merck, Milan, Italy). The homogenates were treated with Laemmli buffer, and 30 µg of proteins were subjected to SDS polyacrylamide gel electrophoresis (4–20% Mini-PROTEAN TGX Stain-Free Gels, Bio-Rad, Segrate, Italy) and electroluted onto the PVDF Membrane (Trans-Blot Turbo Transfer Pack, #1704156, Bio-Rad, Segrate, Italy) with the Trans-Blot Turbo Transfer System (#1704150, Bio-Rad, Segrate, Italy). The membranes were incubated at RT for 1 h with a blocking solution containing 5% skimmed dry milk and 0.1% Tween in tris-buffered saline. Then, the blots were incubated overnight with the blocking solution containing anti-Sigma1 Receptor (B-5) (sc-137075, 1:500 dilution; Santa Cruz Biotechnology, Inc., Heidelberg, Germany). After 3 washings for 10 min at RT, the blots were incubated at RT for 1 h with rabbit anti-mouse antibody (Dakocytomation, P0260, Agilent, Cernusco sul Naviglio, Italy) and diluted 1:100,000 in blocking solution. A Westar Supernova Western blotting detection system (CYANAGEN, Bologna, Italy) was used to visualize the bands, whose molecular weights were measured using pre-stained markers (ab116028, Abcam, Cambridge, UK). As housekeeping, we used the anti-β-2-microglobulin rabbit monoclonal antibody (ab75853, 1:10,000; Abcam, Cambridge, UK). Finally, protein band detection and semiquantitative analysis of the bands were carried out by the iBright™ CL1000 Imaging System and software (Thermo Fisher Scientific, Monza, Italy). The amount of S1R expressed in knocked-down and control HeLa cells was shown as the S1R/β-2-microglobulin ratio.

### 2.5. HyPer7 Hydrogen Peroxide Indicators Transfection for Optical Imaging

Three plasmids were used to express the ultrasensitive hydrogen peroxide indicator HyPer7 in the cytoplasm (pCS2+HyPer7-NES), in the mitochondrial intermembrane space (pCS2+IMS-HyPer7), and in the mitochondrial matrix (pCS2+MLS-HyPer7). The plasmids were a generous gift from Vsevolod Belousov (IBCh, Moscow, Russia) (Addgene plasmid # 136467; Available online: https://n2t.net/addgene:136467; RRID: Addgene_136467; Addgene plasmid # 136469; Available online: https://n2t.net/addgene:136469; RRID: Addgene_136469; Addgene plasmid # 136470; Available online: https://n2t.net/addgene:136470; RRID: Addgene_136470) (accessed on 11 June 2025) [30]. Transfection was performed on HeLa cells at 60–70% confluency in 2 mL Petri dishes as briefly described below. Transfection solution was prepared by adding 2 μg DNA HyPer7 indicator to 200 μL JetOPTIMUS buffer (# 717-60, Polyplus transfection, Illkirch-Graffenstaden, France) and 2 μL JetOPTIMUS DNA Transfection Reagent (# 117-15, Polyplus transfection, Illkirch-Graffenstaden, France) and left for 10 min at RT. Then, this solution was mixed with 2 mL Opti-MEM™ and added to the cells. After 4 h at 37 °C, the transfection medium was substituted with a complete DMEM. Cells were used for imaging experiments 24 h after transfection. When the experiments were performed with HeLa cells with reduced AQP expression, Hyper7 was transfected one day after the knockdown of AQP3, 6, and 11, while for AQP8-knockdown (AQP8-KO) cells, the transfection was performed the day before the knockdown. The experiments were performed at different times after knockdown, 48 h vs. 24 h, because the Western blot experiments to confirm the effectiveness of the knockdown revealed a different turnover time of the AQP proteins.

### 2.6. Detection of H_2_O_2_ in Cytoplasmic and Mitochondrial Compartments by HyPer7 Imaging

The H_2_O_2_ permeability was measured by Hyper7 oxidation with a ratiometric method [30]. Images were collected every 1–2 s for 1 to 5 min by using a Leica TCS SP8 DLS confocal microscope (Leica biosystems, Buccinasco, Italy) with dual excitation at 420 nm and 490 nm and the emission at 530 nm. Since the results obtained by ratiometric estimations were comparable to those acquired by assessing the fluorescence of the HyPer7 biosensors excited at 490 nm and the emission collected at 530 nm, the latter method was routinely utilized.

HyPer7-transfected cells were washed and incubated at RT for 10 min with the following solution: 140 mM NaCl, 5 mM KCl, 2 mM CaCl_2_, 1 mM MgCl_2_, 10 mM D-glucose, and 1 mM HEPES, pH 7.4.

To visualize the fluorescence of transfected cells, the HyPer7-NES, -IMS, and -MLS were excited at 490 nm, and the emitted light was detected at 530 nm. Images were detected using an Olympus BX41 microscope with a 60x water immersion objective (LUMPlanFI 60x/0.90 w, Olympus; Olympus Italia S.r.l., Segrate, Italy) equipped with a CCD camera (DMK 33UP1300; THE IMAGING SOURCE, Visionlink, Seregno, Italy) and collected at 10 fps by IC capture software (v. 2.5). H_2_O_2_ was added to the cells to achieve a final concentration of 50 μM. Images were analyzed utilizing Image J 1.54f software [31]. The time course of H_2_O_2_ diffusion was then analyzed using a one-phase association equation, and the relative initial rate constant (k) was obtained using GraphPad Prism (4.00, 2003).

### 2.7. Double Immunofluorescence

Immunolocalization of AQP3, 6, 8, and 11 and the mitochondrial marker TOMM20 (mitochondrial import receptor subunit) was performed using sub-confluent HeLa cells grown on 18 mm × 18 mm glass coverslips.

Cells were fixed in 4% paraformaldehyde in PBS for 30 min in a Petri dish and then were washed in PBS. Antigen retrieval was performed by boiling the coverslips for 10 min in petri dishes containing retrieval buffer (10 mM citrate–HCl, pH 6.0). Then, the coverslips were blocked with 3% BSA in PBS at RT for 30 min. Successively, the coverslips were incubated overnight at 4 °C with the antibodies anti-AQP and anti-TOMM20. The antibodies used were the following: affinity pure anti-AQP3 rabbit antibody (ab125045, 1:200 dilution; Abcam, UK), anti-AQP6 rabbit polyclonal IgG (AQP61-A, 1:500 dilution; Alpha Diagnostic International, San Antonio, TX, USA), anti-AQP8 rabbit antibody (PA1511, 1:200 dilution; BOSTER, Pleasanton, CA, USA), anti-AQP11 rabbit antibody (PB10044, 1:100 dilution; BOSTER, Pleasanton, CA, USA), and anti-TOMM20 [4F3] (ab56783, 1:500 dilution; Abcam, UK). Three 10 min washes with PBS were performed before incubation at RT for 30 min with the following fluorescent secondary antibodies: Alexa Fluor^®^ 488 AffiniPure^®^ Fab Fragment Goat Anti-Mouse IgG (H+L) (115-547-003, 1:500 dilution; Jackson ImmunoResearch Europe Ltd., St. Thomas Place, UK) and Rhodamine (TRITC) AffiniPure^®^ Goat Anti-Rabbit IgG (H+L) (111-025-045, 1:200 dilution; Jackson ImmunoResearch Europe Ltd., St. Thomas Place, UK). Nuclei were counterstained with Hoechst (14533, Merck Life Science S.r.l., Milano, Italy) and washed thrice with PBS.

The coverslips were mounted with FluoroSave reagent (345789, Merck Life Science S.r.l., Milano, Italy) and examined with a Leica TCS SP8 STED 3X confocal microscopy system (Leica biosystems, Buccinasco, Italy) equipped with an HC PL APO CS2 63x 1.4 NA oil-immersion objective and a 2x zoom. Images were analyzed by LAS AF Lite software (Leica Microsystems Application Suite Advanced Fluorescence Lite version 2.6.0, Buccinasco, Italy). Cells incubated with nonimmune serum were negative controls.

### 2.8. Statistics

Data were reported as means ± SD (standard deviation) of 3–6 different experiments. In each live imaging experiment, fluorescence variations were analyzed in more than 20 cells per condition. The significance of the differences of the means was assessed using Student’s *t*-test and ANOVA, followed by Newman–Keuls’s *Q* test (GraphPad Prism 4.00, 2003).

## 3. Results

### 3.1. AQP3, AQP6, AQP8, and AQP11 Facilitate the Diffusion of H_2_O_2_ Through the Plasma and Mitochondrial Membranes

This study aims to evaluate the possible regulation of S1R on the AQP-mediated permeability to H_2_O_2_ through the plasma membrane and the mitochondrial compartments. In this and previous studies, we found that HeLa cells expressed the following peroxiporins at both mRNA and protein levels: AQP3, AQP6, AQP8, and AQP11 ([10,23,24] Figure S1).

**Figure 1:**
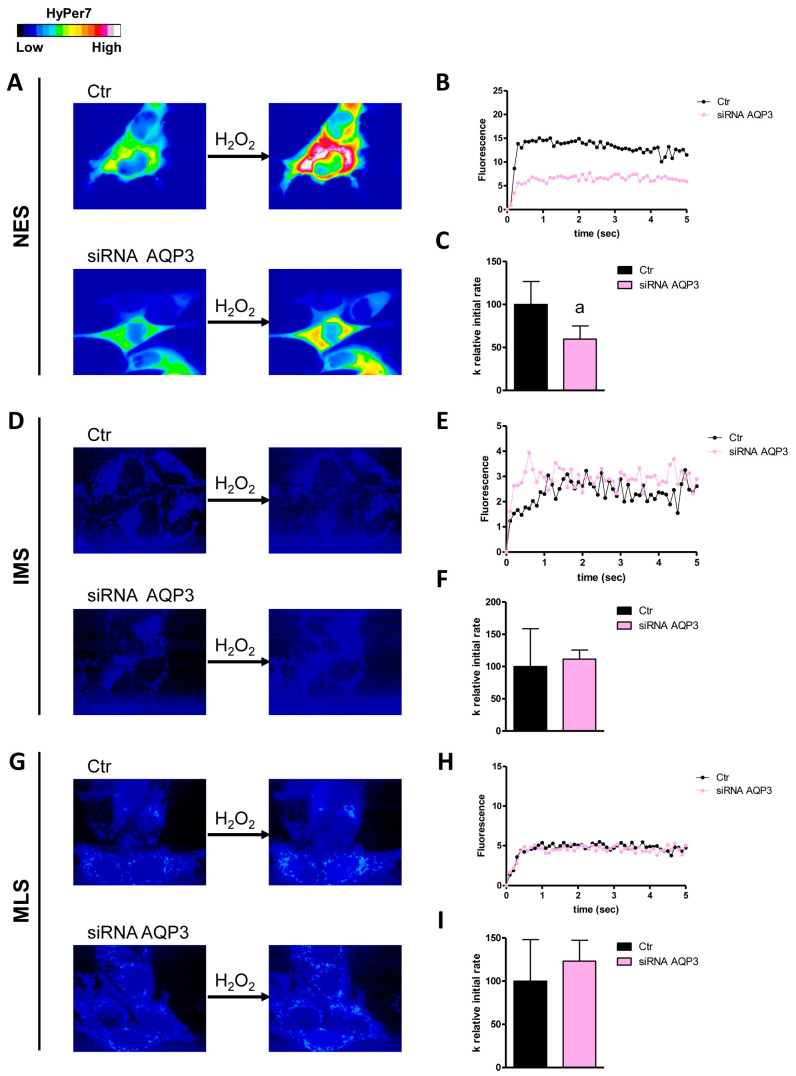
Effect of AQP3 knockdown on hydrogen peroxide diffusion through the plasma membrane (NES), the outer mitochondrial membrane (IMS), and the inner mitochondrial membrane (MLS) of HeLa cells. (**A**,**D**,**G**) The left and right panels display representative frames extracted from videos illustrating the time course of H_2_O_2_ diffusion into mock-transfected (Ctr) and AQP3-knockdown (siRNA AQP3) HeLa cells before and after the addition of 50 μM H_2_O_2_, respectively. The increase in HyPer7 fluorescence is shown in pseudocolor in the upper panel, with the scale indicated in the insert. (**B**,**E**,**H**) The time course of H_2_O_2_ fluorescence in mock- and siRNA-transfected HeLa cells is presented, starting from the addition of 50 μM H_2_O_2_. Data represent the mean of at least three different experiments, with standard deviations omitted for clarity. (**C**,**F**,**I**) Computerized least squares regression analysis was employed to determine the k relative initial rate values (GraphPad Prism 4.00, 2003). The experimental points of the H_2_O_2_ time course curves were fitted using a one-phase exponential association equation. a, *p* < 0.05 compared to Ctr (Student’s *t*-test).

**Figure 2:**
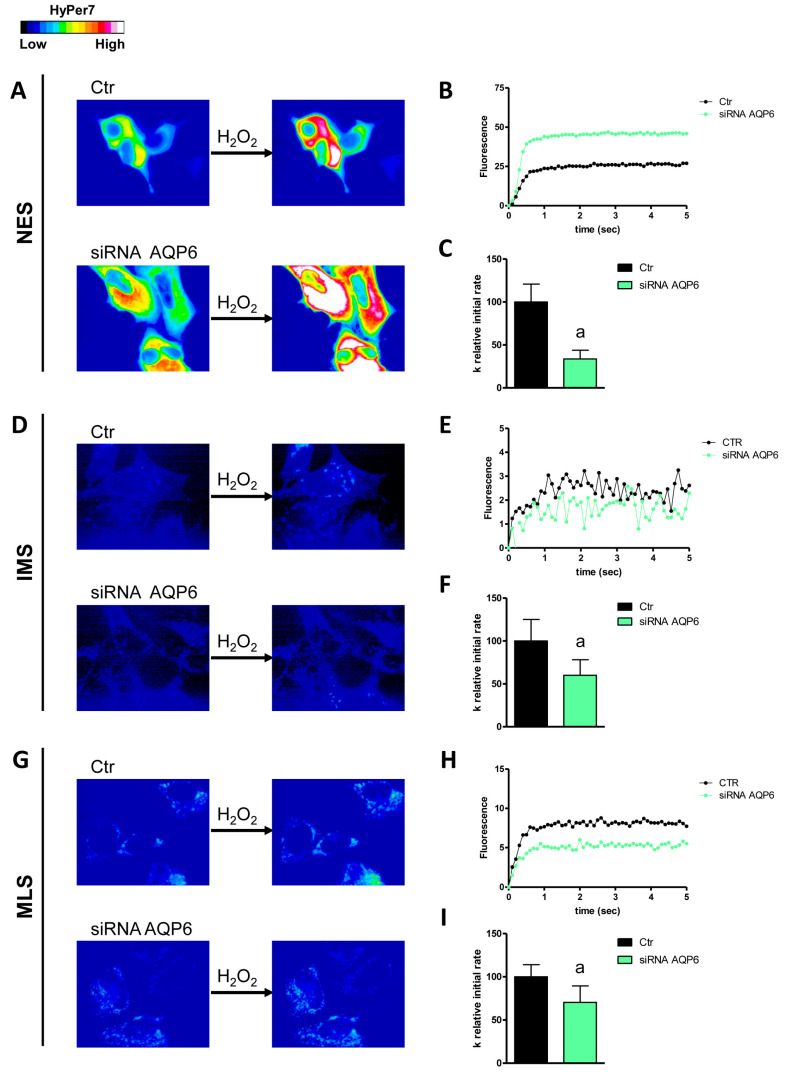
Effect of AQP6 knockdown on hydrogen peroxide diffusion through the plasma membrane (NES), the outer mitochondrial membrane (IMS), and the inner mitochondrial membrane (MLS) of HeLa cells. (**A**,**D**,**G**) The left and right panels display representative frames extracted from videos illustrating the time course of H_2_O_2_ diffusion into mock-transfected (Ctr) and AQP6-knockdown (siRNA AQP6) HeLa cells before and after the addition of 50 μM H_2_O_2_, respectively. The increase in HyPer7 fluorescence is shown in pseudocolor in the upper panel, with the scale indicated in the insert. (**B**,**E**,**H**) The time course of H_2_O_2_ fluorescence in mock- and siRNA-transfected HeLa cells is presented, starting from the addition of 50 μM H_2_O_2_. Data represent the mean of at least three different experiments, with standard deviations omitted for clarity. (**C**,**F**,**I**) Computerized least squares regression analysis was employed to determine the k relative initial rate values (GraphPad Prism 4.00, 2003). The experimental points of the H_2_O_2_ time course curves were fitted using a one-phase exponential association equation. a, *p* < 0.05 compared to Ctr (Student’s *t*-test).

**Figure 3:**
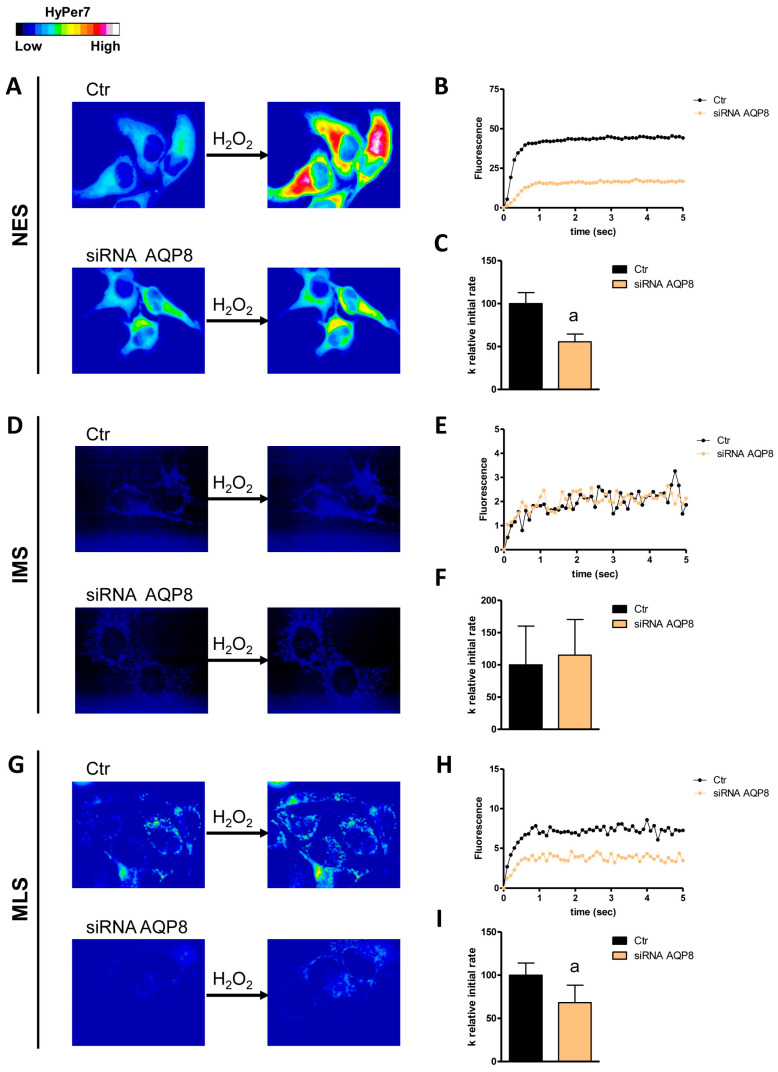
Effect of AQP8 knockdown on hydrogen peroxide diffusion through the plasma membrane (NES), the outer mitochondrial membrane (IMS), and the inner mitochondrial membrane (MLS) of HeLa cells. (**A**,**D**,**G**) The left and right panels display representative frames extracted from videos illustrating the time course of H_2_O_2_ diffusion into mock-transfected (Ctr) and AQP8-knockdown (siRNA AQP8) HeLa cells before and after the addition of 50 μM H_2_O_2_, respectively. The increase in HyPer7 fluorescence is shown in pseudocolor in the upper panel, with the scale indicated in the insert. (**B**,**E**,**H**) The time course of H_2_O_2_ fluorescence in mock- and siRNA-transfected HeLa cells is presented, starting from the addition of 50 μM H_2_O_2_. Data represent the mean of at least three different experiments, with standard deviations omitted for clarity. (**C**,**F**,**I**) Computerized least squares regression analysis was employed to determine the k relative initial rate values (GraphPad Prism 4.00, 2003). The experimental points of the H_2_O_2_ time course curves were fitted using a one-phase exponential association equation. a, *p* < 0.05 compared to Ctr (Student’s *t*-test).

**Figure 4:**
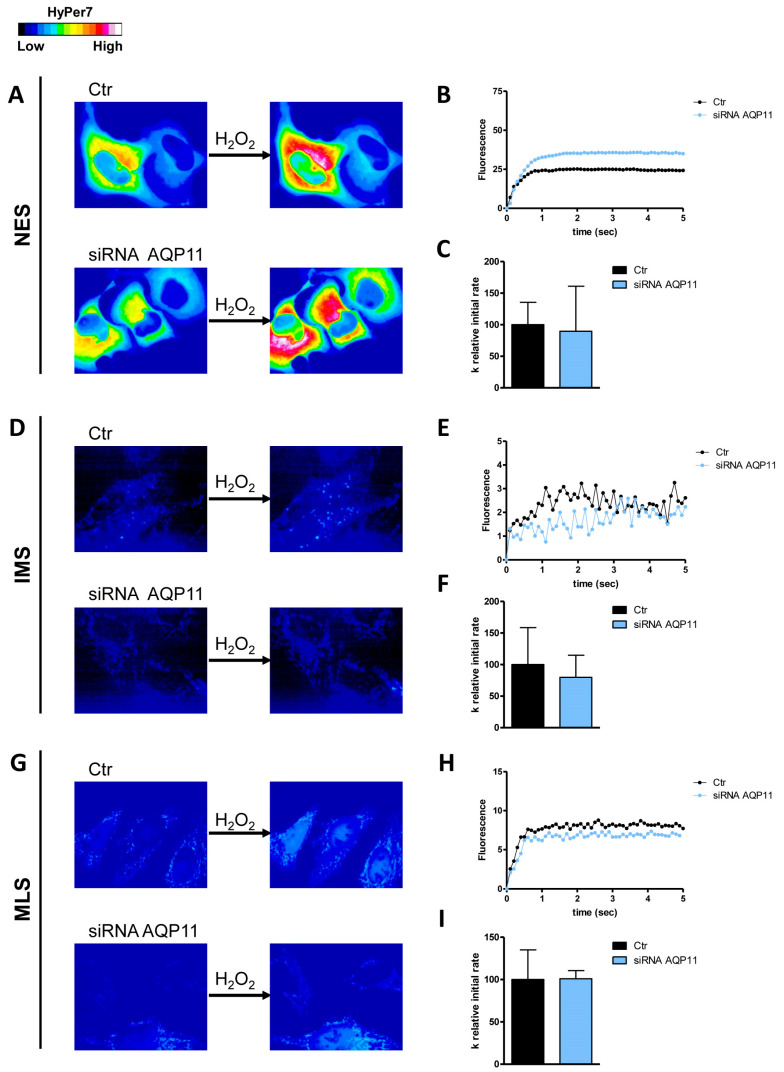
Effect of AQP11 knockdown on hydrogen peroxide diffusion through the plasma membrane (NES), the outer mitochondrial membrane (IMS), and the inner mitochondrial membrane (MLS) of HeLa cells. (**A**,**D**,**G**) The left and right panels display representative frames extracted from videos illustrating the time course of H_2_O_2_ diffusion into mock-transfected (Ctr) and AQP11-knockdown (siRNA AQP11) HeLa cells before and after the addition of 50 μM H_2_O_2_, respectively. The increase in HyPer7 fluorescence is shown in pseudocolor in the upper panel, with the scale indicated in the insert. (**B**,**E**,**H**) The time course of H_2_O_2_ fluorescence in mock- and siRNA-transfected HeLa cells is presented, starting from the addition of 50 μM H_2_O_2_. Data represent the mean of at least three different experiments, with standard deviations omitted for clarity. (**C**,**F**,**I**) Computerized least squares regression analysis was employed to determine the k relative initial rate values (GraphPad Prism 4.00, 2003). The experimental points of the H_2_O_2_ time course curves were fitted using a one-phase exponential association equation.

**Figure 5:**
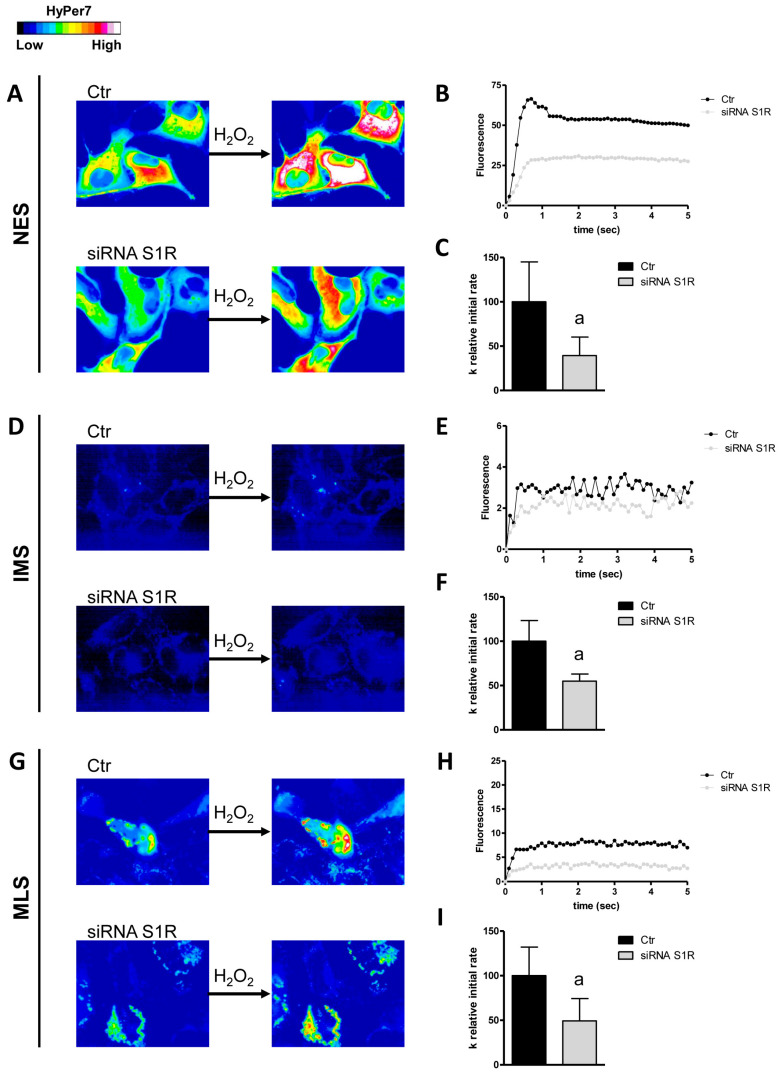
Effect of S1R knockdown on hydrogen peroxide diffusion through the plasma membrane (NES), the outer mitochondrial membrane (IMS), and the inner mitochondrial membrane (MLS) of HeLa cells. (**A**,**D**,**G**) The left and right panels display representative frames extracted from videos illustrating the time course of H_2_O_2_ diffusion into mock-transfected (Ctr) and S1R-knockdown (siRNA S1R) HeLa cells before and after the addition of 50 μM H_2_O_2_, respectively. The increase in HyPer7 fluorescence is shown in pseudocolor in the upper panel, with the scale indicated in the insert. (**B**,**E**,**H**) The time course of H_2_O_2_ fluorescence in mock- and siRNA-transfected HeLa cells is presented, starting from the addition of 50 μM H_2_O_2_. Data represent the mean of at least three different experiments, with standard deviations omitted for clarity. (**C**,**F**,**I**) Computerized least squares regression analysis was employed to determine the k relative initial rate values (GraphPad Prism 4.00, 2003). The experimental points of the H_2_O_2_ time course curves were fitted using a one-phase exponential association equation. a, *p* < 0.05 compared to Ctr (Student’s *t*-test).

The first aim of this study was to understand which AQPs were responsible for the diffusion of H_2_O_2_ across the plasma membrane and the external and internal membranes of the mitochondrion by single gene knockdown of the peroxiporins expressed in HeLa cells. The effectiveness of AQP3, 6, 8, and 11 knockdown has been established and demonstrated by qRT-PCR and Western blot ([24] Figures S2 and S3). The initial level of H_2_O_2_ was not significantly different in the different conditions tested. Moreover, the permeability of the outer mitochondrial membrane was lower compared to that of the inner one (Figure 1, Figure 2, Figure 3, Figure 4 and Figure 5).

AQP3 is an AQP typically expressed at the plasma membrane. In fact, its knockdown resulted in a decreased permeability of the plasma membrane to H_2_O_2_ (about 40% reduction in the k relative initial rate constant) but not of the mitochondrial membranes (Figure 1). HeLa cells with reduced AQP6 showed a significant decrease in H_2_O_2_ diffusion through the outer and inner mitochondrial membranes and through the plasma membrane (Figure 2). An involvement of AQP8 was demonstrated in AQP8-KO cells in either the plasma membrane or the inner mitochondrial membrane, with a reduction of 45% and 32%, respectively (Figure 3).

As for AQP11, its knockdown did not show a decrease in H_2_O_2_ permeability in the plasma membrane and in mitochondrial membranes (Figure 4).

As a whole, the results indicated that AQP3, AQP6, and AQP8 were indeed involved in the diffusion of H_2_O_2_ across the plasma membrane. AQP6 was involved in the diffusion of H_2_O_2_ in the outer mitochondrial membrane, and AQP6 and AQP8 were involved in the diffusion of H_2_O_2_ in the inner mitochondrial membrane.

### 3.2. S1R Modulates the Diffusion of H_2_O_2_ Through the Plasma and Mitochondrial Membranes

The involvement of S1R in the modulation of H_2_O_2_ diffusion through the plasma membrane and the outer and inner mitochondrial membrane has been studied by using knockdown cells for S1R ([10] and Figure S3) and the successive measure of H_2_O_2_ permeability. The results showed that S1R-knockdown (S1R-KO) cells had reduced permeability to H_2_O_2_ with a more pronounced effect at the plasma membrane and inner mitochondrial membrane, with a decrease of 60 and 50%, respectively (Figure 5). Knockdown of S1R caused a significant (about 45%) reduction in H_2_O_2_ diffusion through the outer mitochondrial membrane (Figure 5). This strongly supports the hypothesis of a modulation of peroxiporins mediated by S1R.

### 3.3. S1R Facilitates the Diffusion of H_2_O_2_ Through the Plasma and Mitochondrial Membranes by Modulating AQP6 and AQP8

**Figure 6:**
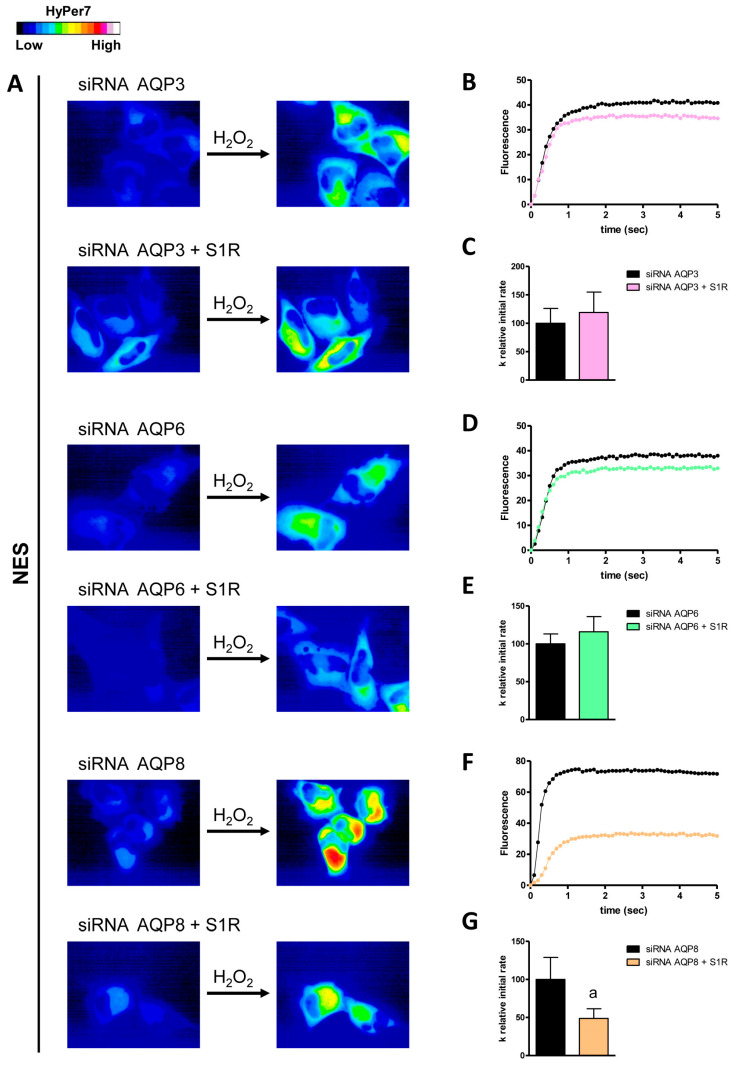
Effect of double knockdown of AQP3/AQP6/AQP8 and S1R in the hydrogen peroxide diffusion through the plasma membrane (NES) of HeLa cells. (**A**) The left and right panels display representative frames extracted from videos illustrating the time course of H_2_O_2_ diffusion into HeLa cells that were single knockdown for AQP (siRNA AQP3/AQP6/AQP8) and HeLa cells that were double knockdown for AQP and S1R (siRNA AQP3/AQP6/AQP8 +S1R) before and after the addition of 50 μM H_2_O_2_, respectively. The increase in HyPer7 fluorescence is shown in pseudocolor in the upper panel, with the scale indicated in the insert. (**B**,**D**,**F**) The time course of H_2_O_2_ fluorescence in HeLa cells with a knockdown of AQP and in cells with a double knockdown of AQP and S1R is presented, starting from the addition of 50 μM H_2_O_2_. Data represent the mean of at least three different experiments, with standard deviations omitted for clarity. (**C**,**E**,**G**) Computerized least squares regression analysis was performed to determine the k relative initial rate values (GraphPad Prism 4.00, 2003). The experimental points from the time courses of H_2_O_2_ curves were fitted using a one-phase exponential association equation. a, *p* < 0.05 compared to control (Student’s *t*-test).

To determine which AQP(s) were regulated by S1R, we performed double-knockdown experiments of S1R alongside single AQPs expressed in the plasma and mitochondrial membranes. In HyPer7-NES-transfected cells, the double knockdown of AQP8 and S1R resulted in a significant decrease in H2O2 permeability (approximately 52%) compared to AQP8-KO cells (Figure 6).

**Figure 7:**
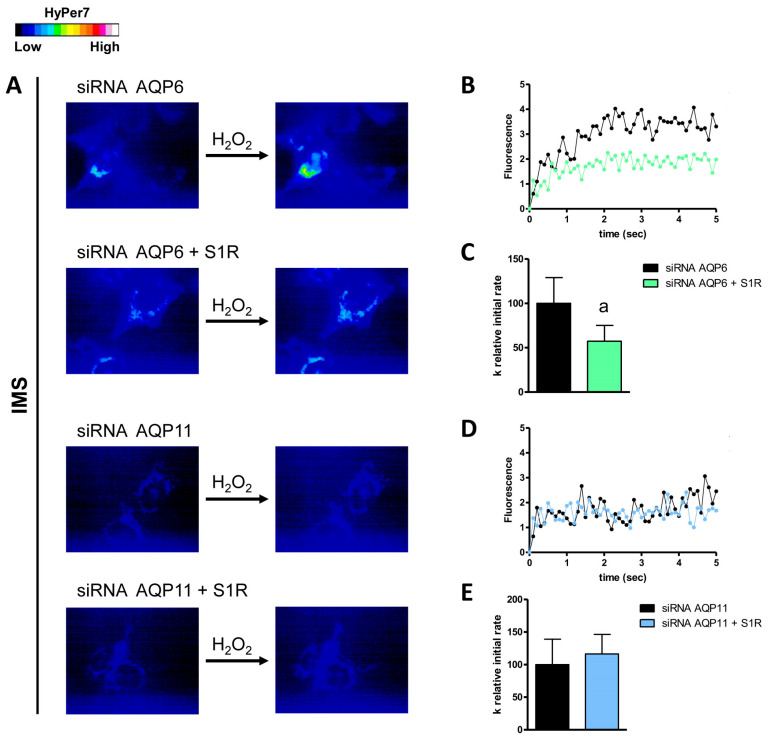
Effect of double knockdown of AQP6/AQP11 and S1R on hydrogen peroxide diffusion through the outer mitochondrial membranes (IMS) of HeLa cells. (**A**) The left and right panels show representative frames extracted from videos displaying the time course of H_2_O_2_ diffusion into HeLa cells that were single knockdown for AQP (siRNA AQP6/AQP11) and HeLa cells that were double knockdown for AQP and S1R (siRNA AQP6/AQP11 + S1R) before and after the addition of 50 μM H_2_O_2_, respectively. The increase in HyPer7 fluorescence is depicted in pseudocolor in the upper panel, with the scale shown in the inset. (**B**,**D**) The time course of H_2_O_2_ fluorescence in HeLa cells knocked down for AQP and in those knocked down for both AQP and S1R is shown, starting from the addition of 50 μM H_2_O_2_. Data are the mean of at least three different experiments, with standard deviations omitted for clarity. (**C**,**E**) Computerized least squares regression analysis was used to determine the k relative initial rate values (GraphPad Prism 4.00, 2003). The experimental points of the time courses of H_2_O_2_ curves were fitted with a one-phase exponential association equation. a, *p* < 0.05 compared to control (Student’s *t*-test).

**Figure 8:**
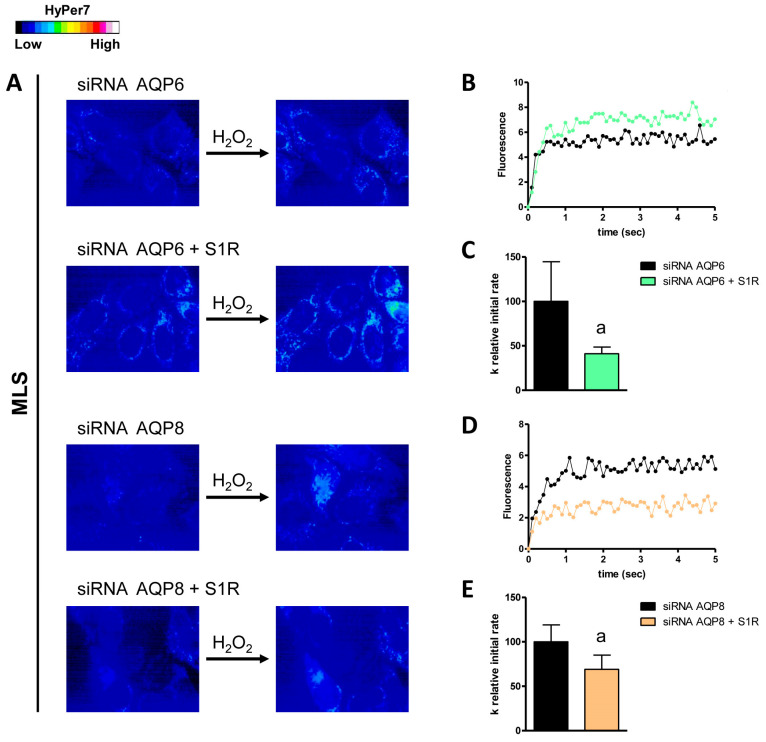
Effect of double knockdown of AQP6/AQP8 and S1R on hydrogen peroxide diffusion through the inner mitochondrial membranes (MLS) of HeLa cells. (**A**) The left and right panels show representative frames extracted from videos displaying the time course of H_2_O_2_ diffusion into HeLa cells that were single knockdown for AQP (siRNA AQP6/AQP8) and HeLa cells that were double knockdown for AQP and S1R (siRNA AQP6/AQP8 + S1R) before and after the addition of 50 μM H_2_O_2_, respectively. The increase in HyPer7 fluorescence is depicted in pseudocolor in the upper panel, with the scale shown in the insert. (**B**,**D**) The time course of H_2_O_2_ fluorescence in HeLa cells knocked down for AQP and in those knocked down for both AQP and S1R is shown, starting from the addition of 50 μM H_2_O_2_. Data represent the mean of at least three different experiments, with standard deviations omitted for clarity. (**C**,**E**) Computerized least squares regression analysis was used to determine the k relative initial rate values (GraphPad Prism 4.00, 2003). The experimental points of the time courses of H_2_O_2_ curves were fitted with a one-phase exponential association equation. a, *p* < 0.05 compared to control (Student’s *t*-test).

Conversely, the double knockdown of AQP3/AQP6 and S1R did not show any significant changes compared to control AQP3-KO and AQP6-KO cells. Regarding the AQPs expressed in the outer mitochondrial membrane, only AQP6 was found to be regulated by S1R. The double knockdown of AQP6 and S1R led to a decrease of about 43% in H_2_O_2_ permeability compared to AQP6-KO cells (Figure 7). Finally, using MLS-HyPer7-transfected HeLa cells, we found that S1R modulated AQP6 and AQP8, as the knockdown of both AQP6/AQP8 and S1R reduced the permeability of the inner mitochondrial membrane by 59% and 31%, respectively (Figure 8).

### 3.4. Mitochondrial Localization of AQPs in HeLa Cells

To confirm the mitochondrial localization of AQP6, AQP8, and AQP11, double immunofluorescence was performed using the mitochondrial marker TOMM20.

**Figure 9:**
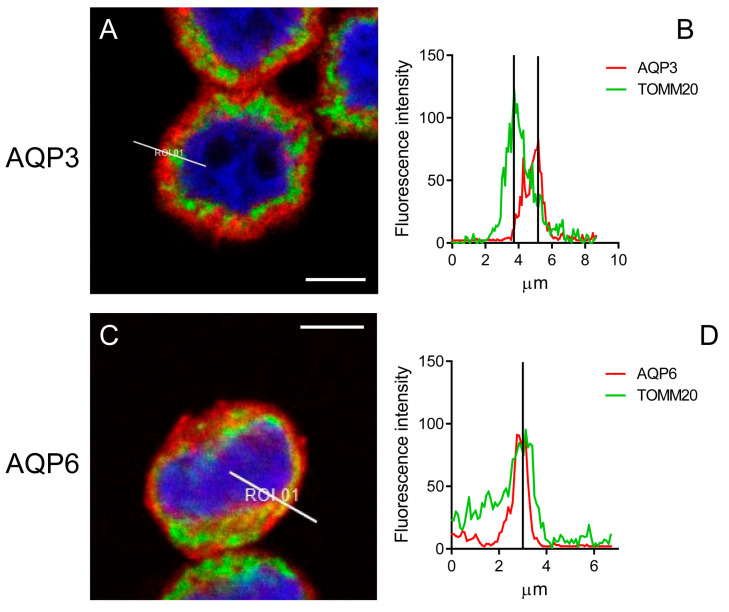
Representative immunofluorescence confocal microscopy images of colocalization of AQP3 and AQP6 with TOMM20 in HeLa cells. (**A**,**C**) Green labeling indicates the presence of TOMM20, and red labeling indicates the expression of AQP3 or AQP6 (Hoechst (blue) counterstained nuclei). Yellow labeling shows colocalization signal of AQP6 with TOMM20. Scale bar, 5 μm. (**B**,**D**) The graph, measured in the white line position in panels (**A**,**C**), shows the fluorescence signals of AQP3 or AQP6 and TOMM20 staining.

**Figure 10:**
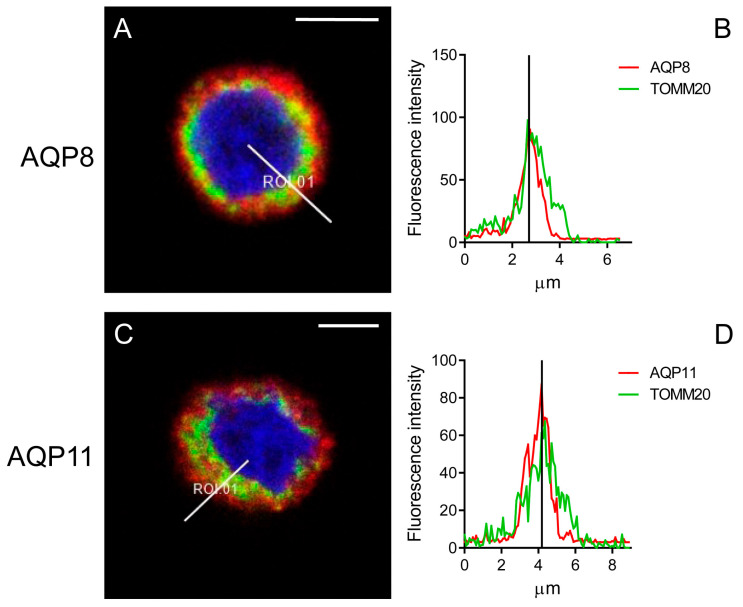
Representative immunofluorescence confocal microscopy images of colocalization of AQP8 and AQP11 with TOMM20 in HeLa cells. (**A**,**C**) Green labeling indicates the presence of TOMM20, and red labeling indicates the expression of AQP8 or AQP11 (Hoechst (blue) counterstained nuclei). Yellow labeling shows colocalization signal of AQP8 or AQP11 with TOMM20. Scale bar, 5 μm. (**B**,**D**) The graph, measured in the white line position in panels (**A**,**C**), shows the fluorescence signals of AQP8 or AQP11 and TOMM20 staining.

Results showed that AQP3, AQP6, and AQP8 are localized in the plasma membrane. However, only AQP6 and AQP8 are also expressed in mitochondria (Figure 9 and Figure 10). The staining for AQP3 and TOMM20 (absence of yellow staining) and the corresponding graph, measured along the white line in panel A, indicated that the fluorescence signals from AQP3 and TOMM20 are separate (Figure 9). Conversely, AQP6 and AQP8 displayed both yellow labeling inside the cells and superficial red labeling, suggesting they are localized in both mitochondria and the plasma membrane (Figure 9C and Figure 10A). The graphs, measured along the white line in Figure 9C and Figure 10A, showed that the fluorescence signals from AQP6 and AQP8 coincided with those from TOMM20 (Figure 9D and Figure 10B), confirming their colocalization with TOMM20.

AQP11 showed only partial colocalization with TOMM20 at specific intracellular points (Figure 10C). At these points, the fluorescence signal graphs for AQP11 and TOMM20 displayed two coincidental peaks (Figure 10D). This is not surprising, considering that AQP11 is expressed in the endoplasmic reticulum, which is closely associated with mitochondria (MAM) [12].

## 4. Discussion

AQPs and S1R have shown antioxidant functions, but only recently has the possibility of their interaction emerged [10]. The antioxidant properties of AQPs are attributable to their ability to regulate H_2_O_2_ diffusion and, therefore, the cellular oxidative state. Eight AQPs, namely AQP0, 1, 3, 5, 6, 8, 9, and 11, are H_2_O_2_ channels, even though all AQPs are virtually permeable to H_2_O_2_ [11,12,13,14,15,16,17,18,19,20,21,22,32]. Given that H_2_O_2_ is the most abundant ROS, the scavenging role of AQPs has acquired a growing interest. For this reason, AQPs were considered promising therapeutic targets for the treatment of diseases characterized by oxidative stress, such as cancer and degenerative disorders.

Studies carried out using ligands have demonstrated the role of S1R in the control of oxidative stress. Cells were protected against ER stress by the overexpression of S1R and by treatment with its agonist, while S1R knockdown and antagonist treatment made cells more sensitive to stress [29].

Recently, we studied the effect of different S1R ligands on water and H_2_O_2_ permeability in the hypothesis that the small molecules already demonstrated to be effective in modulating S1R could also regulate the gating of AQPs [9,10]. The well-established S1R agonists PRE084 and RC33, the antagonist NE100, and three S1R ligands developed by Collina’s Group were tested [26]. The agonists protected HeLa cells from oxidative stress, whereas the S1R antagonist NE100 did not [10]. Experiments performed with HeLa cells knocked down for S1R demonstrated that all the compounds, except for RC33, counteracted the oxidative stress with an antioxidant mechanism mediated by both S1R and AQPs [10]. Interestingly, double-label immunofluorescence experiments showed a partial colocalization and/or contiguity of S1R and AQP3, 8, and 11 [10]. The effect of other S1R agonists and antagonists on AQP permeability was also confirmed in other studies [9].

These results prompted us to investigate the relationship between AQPs and S1R, in particular, to evaluate the possible regulatory function of S1R on AQP-mediated H_2_O_2_ permeability. S1R is expressed in the MAM, and upon activation, S1R can translocate/interact with proteins localized at the mitochondrial and plasma membrane levels [4]. Here, we considered the function of the AQPs expressed in the plasma membrane and the mitochondria. Given that one-third of total cellular H_2_O_2_ is produced by mitochondria, in particular by the mitochondrial matrix, the presence of proteins such as AQPs that facilitate the diffusion of H_2_O_2_ is highly probable and more than a suggestion [33]. Therefore, we studied, by using HyPer7 biosensors, the AQP-mediated H_2_O_2_ diffusion in a three-compartment system: the cytosol, the intermembrane space, and the matrix.

First, we functionally identified the contribution of peroxiporins expressed in the plasma membrane and the outer and inner mitochondrial membranes. The peroxiporins considered were those identified previously [10,24]. AQP3, AQP6, and AQP8 were found in the plasma membrane, AQP6 was found in the outer mitochondrial membrane, and AQP6 and AQP8 were found in the inner mitochondrial membrane.

The functional involvement of AQP3, AQP6, and AQP8 in the plasma membrane was already demonstrated in previous papers [13,24,34].

It is well-recognized that mitochondria change the volume and morphology in response to the water diffusion osmotically driven by the net transport of solutes into and out of the mitochondria, thus suggesting the existence of AQPs [35]. However, data in the literature regarding the existence of functional AQP in mitochondria are conflicting. High water permeability characterizes the inner mitochondrial membrane of rat liver [27]. Immunostaining and immunoblotting experiments showed an AQP9 expression in the inner mitochondrial membrane of the rat brain [36]. However, another study using mitochondria isolated from wild-type and KO mice showed that the diffusion of water and glycerol in the inner mitochondrial membrane takes place with an AQP-independent mechanism [37].

By employing selective single AQP knockdown, we identified AQP6 in the outer mitochondrial membrane as well as AQP6 and AQP8 in the inner mitochondrial membrane.

The functional involvement of AQP8 in the inner mitochondrial membrane is consistent with the results reported previously [27]. Moreover, Calamita et al. [27] suggested the presence of an additional AQP, an Hg^2+^-insensitive one, facilitating the diffusion of water across the inner mitochondrial membrane that we have here identified as AQP6, whose permeability is not inhibited but activated by mercurial compounds [38]. Recently, the involvement of AQP11 in the diffusion of H_2_O_2_ from mitochondria to the ER via AQP11 has been demonstrated [39]. Additionally, Bestetti et al. [12] identified AQP11 not only in the ER but also in small amounts in the MAM. However, in the present study, we show that H_2_O_2_ permeability through the outer and inner mitochondrial membranes is independent of AQP11, as its knockdown had no significant effects on IMS- and MLS-HyPer7 activation [39].

Remarkably, AQP6 is the common thread of H_2_O_2_ diffusion into and out of the external fluid and from the cytosol to mitochondria and vice versa. This may be important because oxidative stress is associated with a decrease in cytosolic and mitochondrial acidification [40,41] that, in turn, can activate AQP6 gating and promote H_2_O_2_ diffusion.

The results of this study, based on immunolocalization experiments using the mitochondrial marker TOMM20, demonstrate that AQP6 and AQP8 are expressed both in mitochondria and in the plasma membrane. The staining of AQP11 appears to partially overlap with that of TOMM20, as indicated by the yellow coloration. Its partial localization in the MAM makes precise localization challenging, which can only be definitively demonstrated through immunogold experiments and electron microscopy.

The second aim of this study was to evaluate the possible control of S1R on the H_2_O_2_ scavenging function of peroxiporins in eustress conditions. Unfortunately, we could not perform the study under oxidative stress conditions because high concentrations of H_2_O_2_ inhibit the permeability of AQPs to H_2_O_2_ and water [13]. First, we proved the involvement of S1R in the modulation of the overall H_2_O_2_ permeability of the plasma membrane and the outer and inner mitochondrial membranes by using S1R-KO cells. Then, we conducted double-knockdown experiments of S1R along with individual AQPs. The results highlighted a regulatory effect of S1R on AQP8 in the plasma membrane and inner mitochondrial membrane and of AQP6 in the outer and in the inner ones. Unfortunately, the effect of S1R on AQP6 in the plasma membrane cannot be demonstrated because of the compensatory upregulation of AQP8 [24].

The modulation by S1R on AQPs has been previously suggested by the results of studies evaluating the effect of S1R agonists and antagonists on AQP permeability [9,10].

Hydrogen peroxide at low physiological concentrations is a signaling molecule [42]; therefore, it is not surprising that S1R can also modulate H_2_O_2_ through the gating of AQPs, given that S1R is recognized as a modulator of other signaling pathways, such as G protein-coupled receptor and ion channel signaling [4]. Moreover, S1R has been shown to influence and interact with more than 49 proteins, sometimes by interaction/translocation with proteins localized in the endoplasmic reticulum itself but also in the mitochondrial, nuclear, and plasma membranes [4].

Despite these findings being observed exclusively in HeLa cells so far, it is highly probable that similar localizations of certain AQPs and S1R regulatory mechanisms are present in other cell types as well. In particular, the expression of AQP3 in the plasma membrane of cells and tissue is well defined. The localization of AQP6 in the plasma membrane was observed in other cell types, such as Met-5A, REN, and MSTO-211H [22], and AQP8 localizes in the plasma membrane of OPM2, Caco-2, and murine I. 29 (unpublished results) and in the inner mitochondrial membranes of rat liver [27]. This is the first report of S1R’s regulatory function on AQP-mediated permeability to H_2_O_2_ despite prior functional data with S1R agonists and antagonists having suggested this relationship [9,10].

## 5. Conclusions

**Figure 11:**
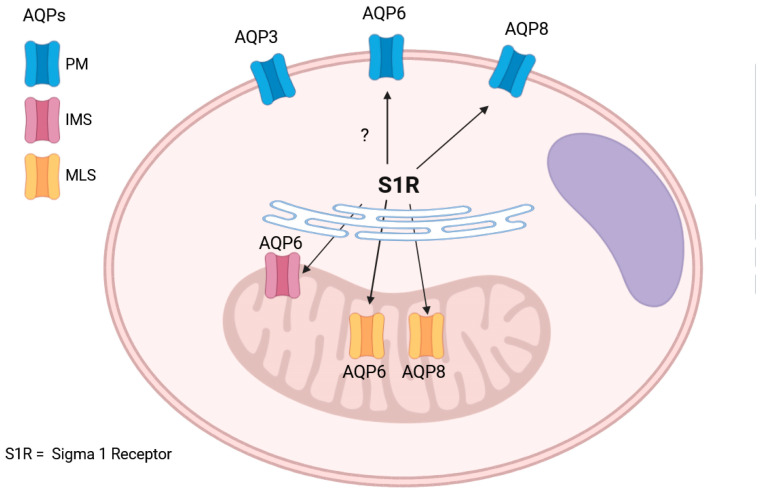
A possible schematic model of the functional AQPs in the plasma membrane and in the outer and inner mitochondrial membranes. AQP3, AQP6, and AQP8 are functionally active in the plasma membrane, AQP6 is functionally active in the outer mitochondrial membrane, while AQP6 and AQP8 are functionally active in the inner mitochondrial membrane. Arrows indicate the modulation of AQP8 by S1R, while the question mark indicates the uncertain modulation of AQP6.

A possible schematic model of the functional AQPs in the plasma membrane and the outer and inner mitochondrial membranes is shown in Figure 11. AQP3, AQP6, and AQP8 are involved in the diffusion of H_2_O_2_ (and water) through the plasma membrane. AQP6 is present in the outer mitochondrial membrane, while AQP6 and AQP8 are present in the inner one, being responsible for the exchange of H_2_O_2_ with cytosol and, probably, endoplasmic reticulum (AQP11). The modulation of AQP8 and AQP6 by S1R, highlighted by arrows, allows for coordination of the H_2_O_2_ movement from the mitochondria to the cytosol and then through the plasma membrane towards the extracellular fluid, favoring the control of the cellular oxidative state.

Although this study provided interesting information about the role of S1R in modulating the function of some peroxiporins, it has some limitations and much more work is needed. Future studies should investigate the physical interaction between S1R and AQPs using protein–protein interaction techniques, such as co-immunoprecipitation or proximity ligation experiments. Unfortunately, it has not been possible to fully clarify the role of individual AQPs due to the existence of a compensatory mechanism, as previously demonstrated. Specifically, the knockdown of AQP6 in HeLa cells led to an increase in the expression of AQP8 [24]. Furthermore, this mechanism of AQP regulation that promotes H_2_O_2_ removal might be common to many cell types. It would be valuable to confirm this in other immortalized cell models as well as in normal and pathological primary cells. Finally, it is important to note that this study was conducted under eustress conditions. A key question that remains is what happens under oxidative stress conditions. Preliminary data suggests that the percentage of colocalization of AQPs with S1R increases significantly under oxidative stress. Consequently, confocal microscopy experiments will be necessary to examine the colocalization of these proteins and assess their potential trafficking under oxidative stress conditions.

## Data Availability

The data supporting the findings of this study are included in the article and its Supplementary Materials.

## Supplementary Materials

The following supporting information can be downloaded at: https://www.mdpi.com/article/10.3390/cells14141082/s1, Table S1: Primer sequences are used for real-time reverse transcription/polymerase chain reaction. Figure S1: Aquaporin -3, -6, -8, and -11 mRNA expression in HeLa cells; Figure S2: Aquaporin -3, -6, -8, and -11 knockdown in HeLa cells; Figure S3: AQP 3 knockdown in HeLa cells; Figure S4: AQP6 knockdown in HeLa cells; Figure S5: AQP8 knockdown in HeLa cells; Figure S6: AQP11 knockdown in HeLa cells; Figure S7: Sigma1 receptor (S1R) knockdown in HeLa cells.
