# Supplementary material for: Sigma1 Receptor Modulates Plasma Membrane and Mitochondrial Peroxiporins
Source: Cells. 2025 Jul 15;14(14):1082. doi: 10.3390/cells14141082 (PMC12293570; doi:10.3390/cells14141082)
Supplement: Supplementary file 1 [file cells-14-01082-s001.zip › cells-3726828-supplementary.pdf]

Table S1. Primer sequences are used for real-time reverse transcription/polymerase chain reaction.

Gene	Primer sequences		Size (bp)	Accession number
AQP3[a]	Forward	5′-CCTGGTGATGTTTGGCTGTGGCTC-3′	147	NM_004925; variants 1, 2
	Reverse	5′-TTCAGGTGGGCCCCAGAGACC-3′		
AQP6	Hs_AQP6_1_SG QuantiTect Primer Assay QT00010633, Qiagen		129	NM_ 001652, XM_006719375
AQP8	Forward	5′-TGGAGAGATAGCCATGTGTGAG-3′	106	NM_001169
	Reverse	5′-TGGCTGCACAAACCGTTCGT-3′		
AQP11	Forward	5′-TTTCTCTTCCACAGCGCTCT-3′	115	NM_173039; variant 1
	Reverse	5′-CTCCTGTTAGACTTCCTCCTGC-3′		
B2M	Hs_B2M_1_SG QuantiTect Primer Assay QT00088935, Qiagen		98	NM_004048
Melting temperature, 60°C; [a], 66°C				

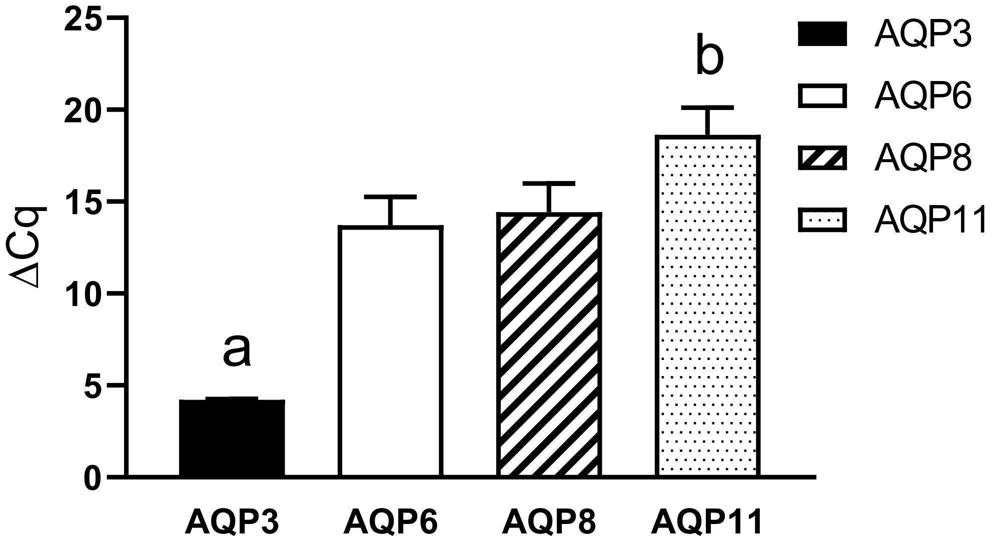

Figure S1. Aquaporin-3, -6, -8, -11 mRNA expression in HeLa cells. AQP mRNA levels were measured by real-time RT-PCR relative to the housekeeping gene β-2-microglobulin. The order of transcript expression levels was as follows: AQP3 showed the highest, followed by AQP6, AQP8, and AQP11. Bars represent the mean ± SD of ΔCq values (n = 4). a, $p < 0.05$ versus AQP6, AQP8, AQP11; b, $p < 0.05$ versus AQP6, AQP8 (ANOVA, followed by Newman-Keuls's *Q* test).

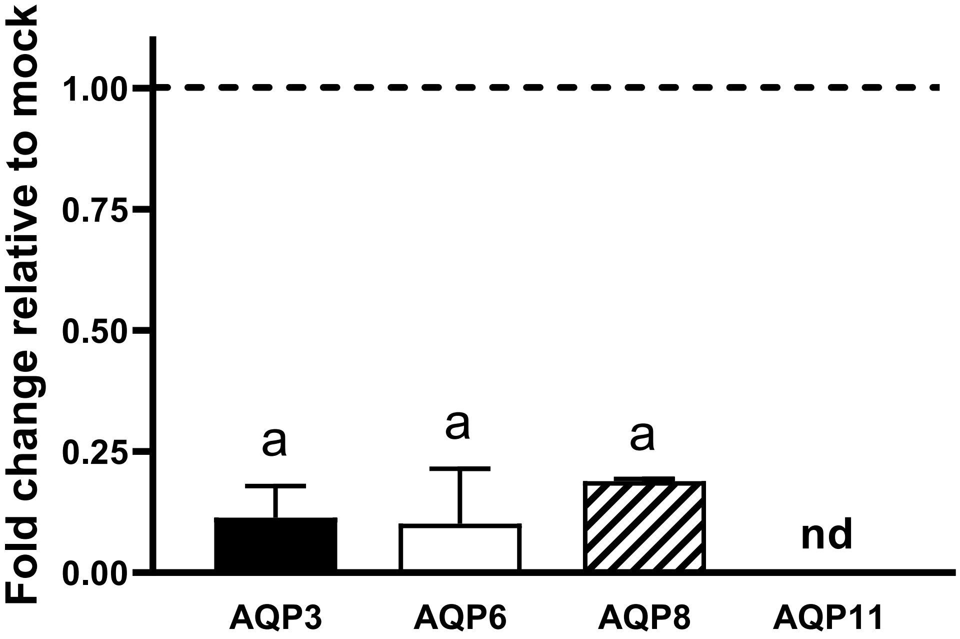

Figure S2. Aquaporin-3, -6, -8, and -11 knockdown in HeLa cells. Mock-transfected and knockdown cells were obtained as described in Materials and Methods. AQP mRNA levels were measured by real-time RT-PCR relative to the housekeeping gene β-2-microglobulin. Bars represent the mean ± SD of the fold change from four different experiments, each using different RNA samples. Knockdown cells showed a reduced RNA content compared to the mock-transfected controls. The dotted line indicates the RNA level of the controls. Statistical analysis was performed by comparing the ΔCq values of knockdown cells and mock cells (controls). a, $p<0.001$ vs. Ctr (Student's *t* test).

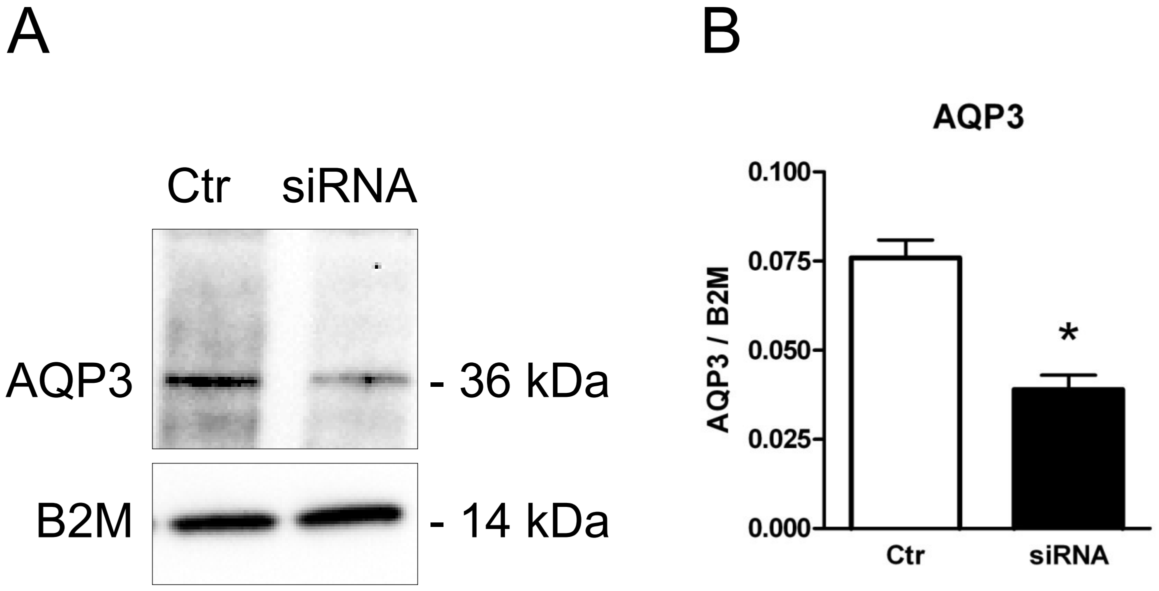

Figure S3 – AQP3 knockdown in HeLa cells. The efficiency of AQP3 knockdown was assessed by immunoblotting and densitometry. Results show a significant reduction in protein expression (50% decrease) in knockdown (siRNA) cells compared to controls (mock-transfected; Ctr). (**A**) A representative blot is shown. Thirty micrograms of protein were loaded, and the blots were incubated with an anti-AQP3 antibody. The blots were then stripped and re-probed with an anti-β microglobulin (B2M) antibody. Bands of 36 kDa and 14 kDa were observed for AQP3 and B2M, respectively. (**B**) Densitometry and semi-quantitative analysis were performed by acquiring the blot with the iBright CL1000 imaging system and iBA (iBright Analysis Software). Results were normalized to B2M. *, $p<0.05$ vs. Ctr (Student's *t* test).

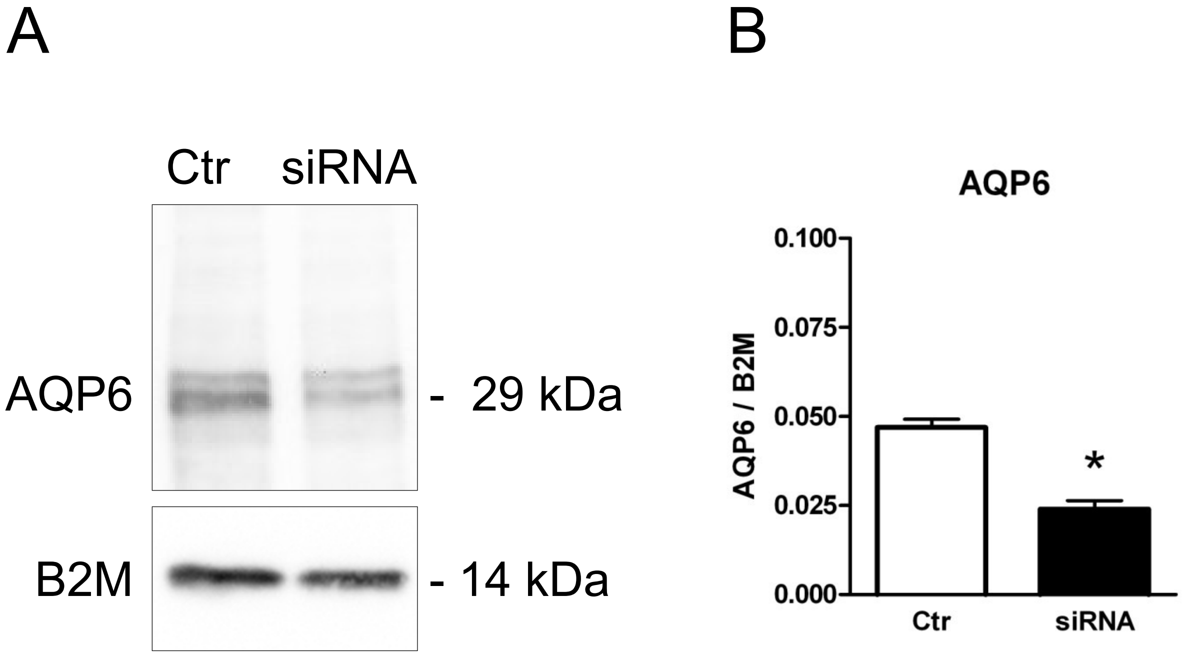

Figure S4 – AQP6 knockdown in HeLa cells. The efficiency of AQP6 knockdown was assessed by immunoblotting and densitometry. Results show a significant reduction in protein expression (50% decrease) in knockdown (siRNA) cells compared to controls (mock-transfected; Ctr). (**A**) A representative blot is shown. Thirty micrograms of protein were loaded, and the blots were incubated with an anti-AQP6 antibody. The blots were then stripped and re-probed with an anti-β microglobulin (B2M) antibody. Bands of 29 kDa and 14 kDa were observed for AQP6 and B2M, respectively. (**B**) Densitometry and semi-quantitative analysis were performed by acquiring the blot with the iBright CL1000 imaging system and iBA (iBright Analysis Software). Results were normalized to B2M. *, $p<0.05$ vs. Ctr (Student's *t* test).

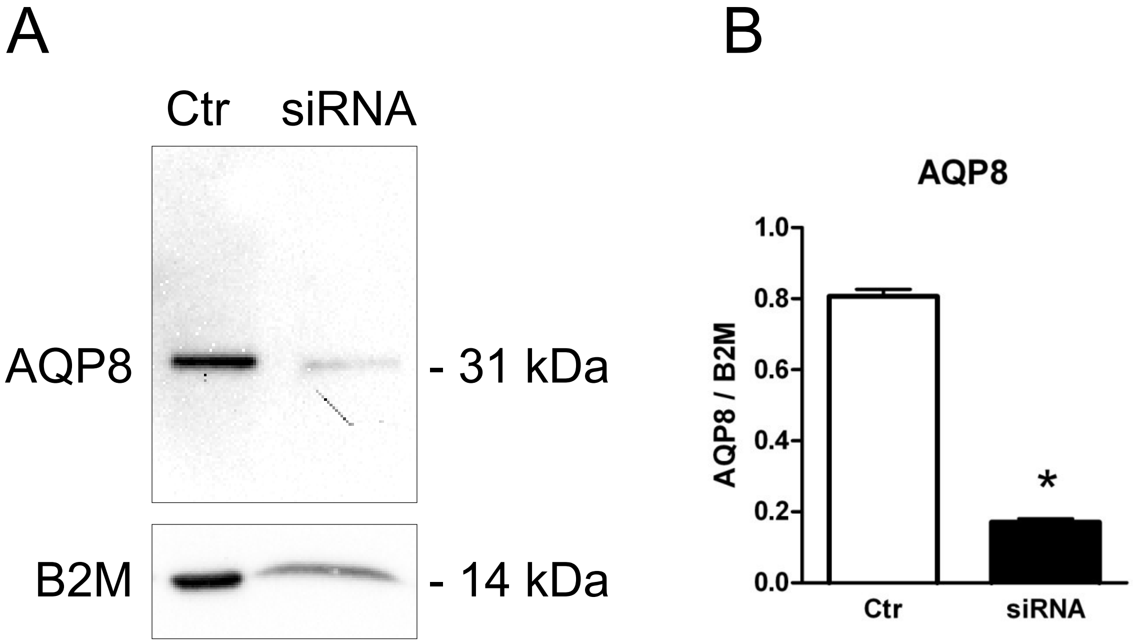

Figure S5 – AQP8 knockdown in HeLa cells. The efficiency of AQP8 knockdown was assessed by immunoblotting and densitometry. Results show a significant reduction in protein expression (79% decrease) in knockdown (siRNA) cells compared to controls (mock-transfected; Ctr). (**A**) A representative blot is shown. Thirty micrograms of protein were loaded, and the blots were incubated with an anti-AQP8 antibody. The blots were then stripped and re-probed with an anti-β microglobulin (B2M) antibody. Bands of 31 kDa and 14 kDa were observed for AQP8 and B2M, respectively. (**B**) Densitometry and semi-quantitative analysis were performed by acquiring the blot with the iBright CL1000 imaging system and iBA (iBright Analysis Software). Results were normalized to B2M. *, $p<0.05$ vs. Ctr (Student's *t* test).

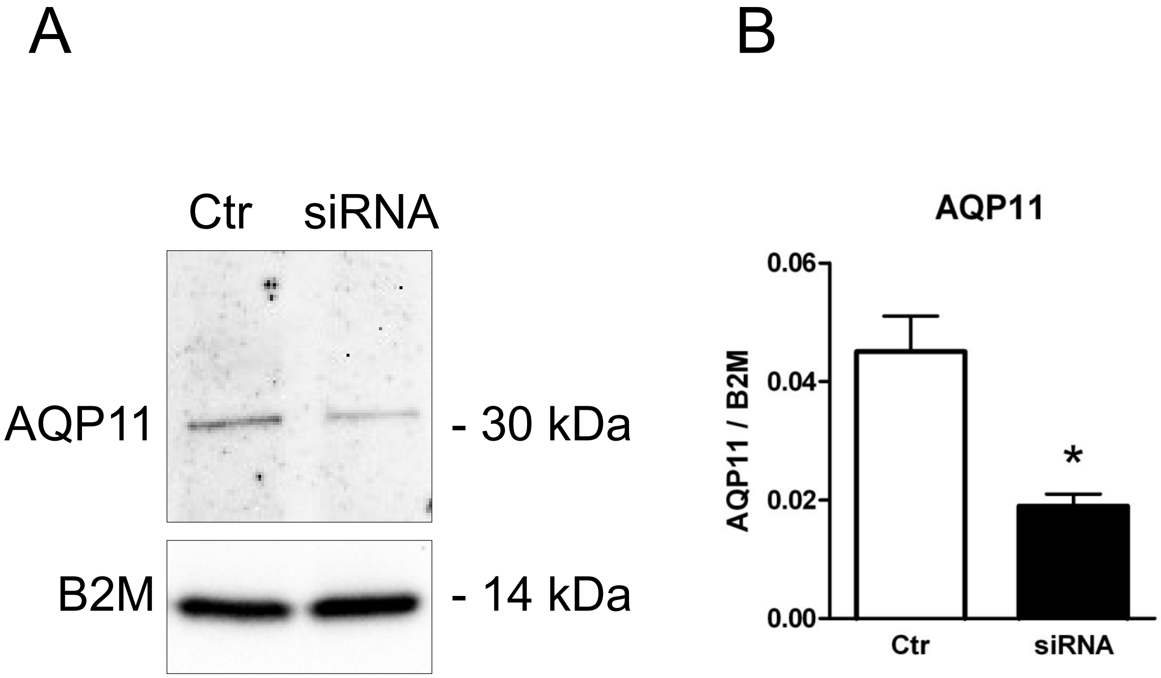

Figure S6 – AQP11 knockdown in HeLa cells. The efficiency of AQP11 knockdown was assessed by immunoblotting and densitometry. Results show a significant reduction in protein expression (58% decrease) in knockdown (siRNA) cells compared to controls (mock-transfected; Ctr). (**A**) A representative blot is shown. Thirty micrograms of protein were loaded, and the blots were incubated with an anti-AQP11 antibody. The blots were then stripped and re-probed with an anti-β microglobulin (B2M) antibody. Bands of 30 kDa and 14 kDa were observed for AQP11 and B2M, respectively. (**B**) Densitometry and semi-quantitative analysis were performed by acquiring the blot with the iBright CL1000 imaging system and iBA (iBright Analysis Software). Results were normalized to B2M. *, $p<0.05$ vs. Ctr (Student's *t* test).

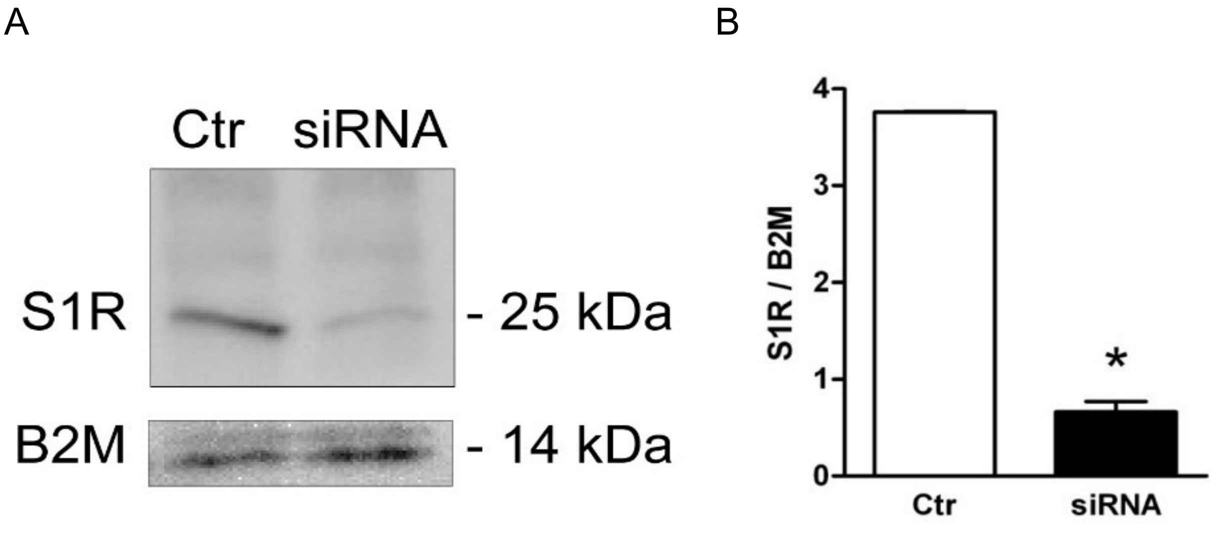

Figure S7 – Sigma1 receptor (S1R) knockdown in HeLa cells. The efficiency of S1R knockdown was assessed by immunoblotting and densitometry. Results show a significant reduction in protein expression (82% decrease) in knockdown (siRNA) cells compared to controls (mock-transfected; Ctr). (**A**) A representative blot is shown. Thirty micrograms of protein were loaded, and the blots were incubated with an anti-S1R antibody. The blots were then stripped and re-probed with an anti-β microglobulin (B2M) antibody. Bands of 25 kDa and 14 kDa were observed for S1R and B2M, respectively. (**B**) Densitometry and semi-quantitative analysis were performed by acquiring the blot with the iBright CL1000 imaging system and iBA (iBright Analysis Software). Results were normalized to B2M. *, $p<0.001$ vs. Ctr (Student's *t* test).
